# Arsenic Stress Resistance in the Endophytic Fungus *Cladosporium cladosporioides*: Physiological and Transcriptomic Insights into Heavy Metal Detoxification

**DOI:** 10.3390/jof11050374

**Published:** 2025-05-14

**Authors:** Xiao-Xu You, Xiao-Gang Li, Xing-Kai Zhang, Wen Gu, Di Chen, Sen He, Guan-Hua Cao

**Affiliations:** 1 School of Chinese Materia Medica and Chinese Pharmaceutical Research International Science and Technology Cooperation Base of Yunnan University of Chinese Medicine, Kunming 650500, China; youxiaoxu2023@163.com (X.-X.Y.); amazing0428@163.com (X.-G.L.); 18469103568@163.com (W.G.);; 2 State Key Laboratory for Quality Ensurance and Sustainable Use of Dao-Di Herbs, Beijing 100700, China; 3 Chinese Pharmaceutical Research International Science and Technology Cooperation Base of Yunnan University of Chinese Medicine, Kunming 650500, China; 4 Wenshan Institute of Food and Drug Control, Wenshan 663099, China

**Keywords:** Cladosporium cladosporioides, endophytic, arsenic (As), arsenic reduction, oxidative stress damage

## Abstract

This study aims to evaluate the tolerance of an endophytic fungus isolated from the fibrous roots of *Gentiana yunnanensis* Franch. to arsenic (As) and elucidate the underlying physiological and molecular mechanisms. The filamentous fungus is identified as *Cladosporium cladosporioides* based on morphological characteristics and phylogenetic tree analysis, belonging to the family Moniliaceae and Phyla Hyphomycetes. The tolerance of *C. cladosporioides* to As(V) was assessed by measuring its biomass under varying concentrations of As(V). The fungus exhibited remarkable As(V) tolerance, with an EC_50_ value of 2051.94 mg/L, and accumulated high concentrations of As in its mycelium. Subcellular distribution analysis revealed that As was predominantly localized in the cell wall fraction, with levels 4.06 times higher than those in the non-cell wall fraction. Notably, the concentrations of total organic As and As(III) in the mycelium were 852.75 μg/g and 24.94 μg/g, respectively, with conversion ratios of 76.64% and 2.24%. The organic As levels significantly surpassed both As(V) and As(III) concentrations in all cellular fractions (cell wall and non-cell wall components), demonstrating particularly efficient As transformation in *C. cladosporioides*. Under As(V) stress, the membrane antioxidant system, including superoxide dismutase (SOD), metallothionein (MT), glutathione (GSH), and melanin, was activated and significantly enhanced to mitigate oxidative damage. Transcriptomic analysis identified 4771 differentially expressed genes (DEGs; 2527 upregulated), including highly expressed As-responsive genes (*CcArsH_1*, *CcARR_1*, *CcARR_*3, *CcGST_1*, and *CcGST*_*3*). Strong correlations emerged between As speciation (total/organic/As(V)/As(III)), antioxidant levels, and DEG expression patterns. Taken together, these findings demonstrate that *C. cladosporioides* employs a multi-faceted As detoxification strategy involving subcellular distribution and reductive transformation (As(V) to As(III)/organic As), antioxidant system enhancement, transcriptomic adaptations, and integrated defense strategy. This work highlights *C. cladosporioides* potential for As bioremediation and elucidates As accumulation mechanisms in *G. yunnanensis*.

## 1. Introduction

*Gentiana yunnanensis* Franch. is a renowned traditional Chinese medicinal herb, utilized for its dried roots and rhizomes in 180 traditional Chinese medicines [1]. It is predominantly found in southwest China, such as in Yunnan Province. Pharmacological studies have found that the primary effective component of *G. yunnanensis* is gentiopicroside, which exhibits potential functions in liver-protective and immune-enhancing properties [2,3]. However, due to the impact of high heavy metal (HM) background levels, such as arsenic (As), in the soil and the surrounding environment, *G. yunnanensis* has occasionally exhibited As levels exceeding the standard limits [4] (1 mg/kg; National Pharmacopoeia of the People’s Republic of China, 2020 edition). Evidence indicates that microorganisms, particularly endophytic fungi, play a significant role in the process of heavy metal enrichment in plants. Their mycelia can absorb and accumulate HMs, and they can also enhance the absorption and accumulation of HMs in host plants [5,6]. Numerous studies have shown that endophytic fungi such as *Burkholderia* spp. and *Pseudomonas* spp., isolated from *Cattleya walkeriana*, can facilitate zinc (Zn) uptake in plants [7]. Additionally, *Alternaria tenuissima* has been found to effectively promote growth and enhance selenium (Se) absorption in its hyperaccumulator host, *Astragalus bisulcatus* [8].

According to the World Health Organization (WHO), HMs, such as As, Cd (cadmium), Pb (lead), and Hg (mercury), are among the most hazardous pollutants. Soil As contamination in China has emerged as a critical environmental challenge, particularly in the *G. yunnanensis* cultivation zones of Yunnan Province. Recent studies have revealed alarming As concentrations of up to 20.9 mg/kg in these soils [9]. These metals are non-degradable, capable of bioaccumulating in the environment, and can transfer through the food chain, posing significant risks to both ecosystems and human health. As, a teratogenic and highly carcinogenic HM, widely exists in soil in the form of As(V), posing significant risks to human health. Long-term exposure to As is associated with an increased risk of various health issues, including cancer, skin lesions, pigmentation changes, cardiovascular diseases, and diabetes [10]. Therefore, elucidating the role of microorganisms in plant As absorption and their tolerance mechanisms holds significant practical importance. Such research can contribute to improving the safety of medicinal plants for both medicinal and edible purposes, as well as facilitating the development of biological methods for remediating As-contaminated soils.

Endophytes are organisms that inhabit healthy plant tissues without causing disease symptoms in their host plants [11]. Among endophytes, endophytic fungi represent a substantial and remarkably diverse group of endophytes, with an estimated one million unique fungal taxa identified [12]. Numerous studies have demonstrated that certain endophytic fungi enhance plants’ ability to tolerate abiotic stress, such as HMs, salinity, and drought. Notably, a significant proportion of these fungi exhibits considerable tolerance to HMs, including As [13]. Evidence demonstrates that endophytic fungi enhance HM uptake and sequestration in plant tissues, thereby reducing HM bioavailability in soil and mitigating toxicity [14]. For instance, El-Mahdy et al. reported that *Aspergillus niger* and *Penicillium chrysosporium* significantly improved Cd and Pb tolerance in *Vicia faba* L. This was achieved through a notable reduction in Cd toxicity and a substantial improvement in plant growth. The underlying mechanisms included the activation of antioxidant systems, the conversion of cadmium into chemically inactive forms, and the redistribution of subcellular Cd to the cell wall [15].

These heavy metal-resistant endophytic fungi demonstrate a remarkable tolerance to metal ions by enhancing the activity of key antioxidant enzymes, such as superoxide dismutase (SOD) and peroxidase (POD), thereby alleviating the damage caused by membrane lipid peroxidation [7]. Additionally, the fungal cell wall plays a crucial role in protecting cells and adsorbing HMs [16]. Composed primarily of polysaccharides and chitin, the fungal cell wall acts as a barrier to metal ions and other solutes, regulating their uptake into the cell [17]. Beyond immobilizing metals on the cell wall, endophytes have developed diverse intracellular mechanisms for metal chelation. Chelating agents within the cytoplasm can deactivate metals, thereby minimizing cellular damage caused by excessive metal intrusion [18]. The reduction in highly reactive metal ions into less toxic inorganic or organic forms is another key mechanism of endophytic fungal detoxification. This has been demonstrated in the *Microbacterium aoyamense* and *Bacillus pseudomycoides* strain [19], which can reduce As(V) to As(III) and organic As compounds. Currently, studying the fundamental molecular mechanisms underlying fungal tolerance and adaptation to HM stress is a significant area of scientific research. The application of transcriptome and proteome sequencing analysis techniques to identify differentially expressed genes (DEGs) or proteins in response to HM induction is widely practiced in current research. Studies have reported that the expression of genes associated with HM transport and detoxification, such as several GST genes identified in *Exophiala pisciphila,* and the gene *LbMT1* in *Laccaria bicolor*, is upregulated following treatments of Pb, Cd, and Cu, respectively [20,21]. However, research on the metabolic pathways and regulatory mechanisms underlying endophytic tolerance to HMs remains limited.

There is a notable scarcity of research that has systematically evaluated and analyzed the HM tolerance mechanisms of endophytic fungi in medicinal plants [22], particularly in species such as *G. yunnanensis,* growing in heavy metal-rich environments. Furthermore, the potential applications of these fungi in mitigating HM accumulation in medicinal plants remain underexplored. In the initial phase of our research, we isolated a heavy metal-resistant endophytic fungus with significant As tolerance from the roots of *G. yunnanensis*. In this study, we aimed to (1) determine the taxonomic classification of this strain and (2) elucidate its As resistance mechanisms by investigating its physiological responses and conducting transcriptome analysis. Our findings not only highlight the potential application of *C. cladosporioides* in the remediation of As-contaminated soils but also provide insights into the mechanisms underlying As accumulation in *G. yunnanensis*.

## 2. Materials and Methods

### 2.1. Material

The filamentous fungi under investigation were isolated from *G. yunnanensis.* The plant samples were collected in Lincang City in September 2022 and were taxonomically identified by Professor Ronghua Zhao.

Surface disinfection and endophytic fungal isolation were conducted following Shah et al. [23]. Root samples were thoroughly rinsed with running water until the complete removal of soil particles and microorganisms was confirmed by clear rinse water. After surface sterilization in 75% ethanol (3–4 min) followed by 5% sodium hypochlorite (5–7 min), samples were rinsed three times with sterile distilled water. Using a sterile technique, roots were aseptically sectioned into 1.5 cm segments and plated on both potato dextrose agar (PDA) and malt extract agar (MEA) supplemented with 1% penicillin–streptomycin. Plates were incubated at 28 °C in complete darkness. Fungal colonies began emerging from root segments by day 3 of incubation. Distinct colonies were subcultured onto fresh antibiotic-free PDA plates for purification. All purified fungal isolates were maintained in sterile test tubes containing PDA slants at 4 °C for long-term storage.

### 2.2. Morphological and Molecular Identification of the Target Endophytic Fungus

The morphological characteristics of the fungi were assessed using pure cultures and coverslip-inserted cultures on a PDA medium, following the methodology outlined by Bensch et al. [24]. Morphological identification was performed based on several criteria, including growth pattern, hyphae structure, colony and medium coloration, surface texture, margin characteristics, aerial mycelium, medium coloration, and the size and pigmentation of conidia, following the identification manuals of the Fungal Identification Handbook [25]. The micromorphological characteristics of the reproductive structures of the isolated strains were observed and photographed on each coverslip using a ZEISS Axio Scope A1 light microscope (Carl Zeiss AG, Oberkochen, Germany) [26].

Molecular identification was carried out through DNA amplification and sequencing of the internal transcribed spacer (ITS) region, following a standard molecular biological protocol. The universal primer pairs ITS1 (5′-TCCGTAGGTGAACCTGCGG-3′) and ITS4 (5′-TCCTCCGCTTATTGATATGC-3′) were utilized to amplify the ITS region via polymerase chain reaction (PCR) [27]. Genomic DNA was extracted from mycelia cultured in modified Melin–Norkrans (MMN) liquid medium for 9 days, and the MMN medium composition included 20 g of glucose, 0.15 g of MgSO_4_, 0.025 g of NaCl, 0.05 g of CaCl_2_, 1.2 mL of FeCl_3_ (1%), 0.5 g of KH_2_PO_4_, 0.25 g of (NH_4_)_2_HPO_4_, 100 μg of vitamin B_1_, and distilled water to make up 1000 mL of solution, adjusted to a pH of 5.8, following the instructions provided with the Microbial DNA Extraction Kit (Bioteke, Beijing, China). The PCR mixture was prepared with 2 μL of genomic DNA template, 9 μL of PCR Master Mix (Fermentase, Burlington, ON, USA), 1 μL of forward primer, 1 μL of reverse primer, and 12 μL of sterile water, yielding a total reaction volume of 25 μL. The PCR cycling protocol consisted of an initial denaturation step at 94 °C for 4 min, followed by 30 cycles of denaturation at 94 °C for 45 s, annealing at 55 °C for 45 s, and extension at 72 °C for 1 min. A final extension step was carried out at 72 °C for 10 min [28]. The amplified DNA products were sequenced by Beijing Tsingke Biotech Co., Ltd., Beijing, China. Sequence similarity searches were conducted using the BLAST+2.16.0 tool on the NCBI database. Homologous fungal ITS region sequences were retrieved from NCBI, and a phylogenetic tree was constructed using the neighbor-joining method with 1000 bootstrap replicates in MEGA software (version 7).

### 2.3. Tolerance of C. cladosporioides to As(V)

The tolerance of *C. cladosporioides* to As(V) was assessed by measuring its biomass under varying concentrations of As(V). Initially, the fungus was activated on PDA plates for two weeks. Six agar blocks, each 0.7 cm in diameter, were excised from the edge of the colony and transferred into 100 mL of MMN. Stock solutions of As(V) were prepared using Na_2_HAsO_4_.7H_2_O and added to the MMN medium to achieve final concentrations of 200, 400, 600, 800, and 1200 mg/L for strain cultivation. Following 9-day cultivation, biomass was harvested, oven-dried at 70 °C until weight stabilization, and measured as dry weight [29]. Each concentration was tested with three replicates. Following the culturing period, the EC_50_ value was calculated by fitting a linear regression model to the biomass inhibition data [30]. The EC_50_ value was then used to evaluate the HM tolerance of *C. cladosporioides*.

### 2.4. Growth Curve of C. cladosporioides

*C. cladosporioides* was cultured in 100 mL MMN medium triangular flasks with or without EC_50_ concentrations of As(V) at 28 ± 1 °C and 120 rpm. Starting from the second day after inoculation, samples were collected daily from at least three flasks, and the mycelium was filtered using pre-dried filter paper. The filtered mycelium was then placed in the oven at 70 °C for 24 h and weighed to plot the growth curve.

### 2.5. Concentration and Distribution of Total As and Different Valence States of As in Mycelium

The total mycelium was collected by suction filtration after being cultured for 1 week at 28 °C and 120 rpm in 100 mL of MMN medium. The mycelium from each triangular flask was thoroughly rinsed three times with 25 mL of 10 mmol/L of ethylene diamine tetraacetic acid (EDTA) to remove any attached metal ions [31]. Subsequently, the contents of total As, As(V), and As(III) and total organic As in mycelium were determined under the treatment of an EC_50_ concentration of As(V). For each treatment group, a dried mycelium sample weighing 0.100–0.300 g was digested with a mixed acid consisting of nitric acid, perchloric acid, and concentrated sulfuric acid (5:1.5:1) at 220 °C for 6–8 h. The digestion solution was then analyzed using a PF6 atomic fluorescence spectrophotometer (Persee, Beijing, China) according to the manufacturer’s instructions. For the determination of As concentration and distribution in the cell wall and non-cell wall fractions, the method described by Teng et al. [32] was followed.

A total of 0.500 g of fresh mycelium sample was weighed and ground into a fine powder. The powder was mixed with a subcellular extraction solution at a ratio of 1:3 (subcellular extraction solution: 50 mM Tris-HCl, 250 mM sucrose, 1 mM dithioerythritol (DTE), pH 7.5). After homogenization, the mixture was transferred to a 50 mL centrifuge tube and centrifuged at 4 °C and 6000 rpm for 10 min. The supernatant and precipitate represented the non-cell wall and cell wall components, respectively. Subsequently, the concentrations of total As, As(V), and As(III) in the non-cell wall and cell wall fractions were determined using the same method described above. Standard solution working curves for total As, As(V), and As(III) were constructed using As standard stock solution, sodium hydrogen arsenate (Na_2_HAsO_4_), and arsenic trioxide (As_2_O_3_), respectively. The respective regression equations were as follows: total As, Y = 0.0028X + 0.0002, R^2^ = 0.9976; As(V), Y = 5.0276X + 18.9904, R^2^ = 0.9996; As(III), Y = 15.1036X − 12.5293, R^2^ = 0.9991. The concentrations in samples were calculated by substituting measured fluorescence values into these standard curves. The total organic As content was calculated as the total As content minus the As(V) content and the As(III) content.

### 2.6. Antioxidant System of C. cladosporioides

A total of 1.000 g of fresh mycelium (cultured in MMN medium as previously described) was weighed and ground in an ice bath with 4 mL of phosphate buffer (pH 7.0), followed by centrifugation at 12,000× *g* and 4 °C for 10 min. SOD activity was determined using a modified SOD Activity Assay Kit (AKAO001M, Beijing Boxbio Co., Ltd., Beijing, China) [33]. Superoxide anion (O^2−^) is produced through the xanthine and xanthine oxidase reaction system, and O^2−^ can reduce nitroblue tetrazolium (NBT) to formazan, which is measured at an absorbance of 560 nm. MDA content was determined using the Kit for MDA Content Detection (AKFA013C, Beijing Boxbio Co., Ltd.). The concentration was estimated using an extinction coefficient by subtracting the nonspecific absorbance at 600 nm from the value determined at 532 nm [34]. The GSH content was determined as described by the Kit for GSH Content Detection (AKPR008C, Beijing Boxbio Co., Ltd.) [35]. Reduced glutathione was used as a substrate to generate the calibration curve at 412 nm. Based on the calibration curve, the absorbance of the sample was used to calculate the sample concentration. The concentration of Pro was determined by extracting free Pro with 3% (*w*/*v*) sulfosalicylic acid and measuring the absorbance at 520 nm, as described by the Kit for Pro Content Detection (AKAM003C, Beijing Boxbio Co., Ltd.) [36]. MT was extracted from fresh mycelium using an ethanol–chloroform method and quantified by enzyme-linked immunosorbent assay (ELISA) [37]. After centrifugation at 6000× *g* for 15 min at 4 °C, the extract was used for MT quantification using the MT ELISA Kit (MM-094101, Meimian Technology, Shanghai, China). Melanin was also extracted from fresh mycelium using an acetone–methanol method and quantified by ELISA [38], and the extract was used for melanin quantification using the Melanin ELISA Kit (MM-9146401, Meimian Technology, China).

### 2.7. Transcriptome Analysis and Availability of Supporting Data

Fresh mycelium treated with or without an EC_50_ concentration of As(V) was used for total RNA extraction. Total RNA isolation, library construction, and sequencing were performed by Metware Biotechnology Co., Ltd. (Wuhan, China). Prior to sequencing, the quality of total RNA was assessed using a Qubit 4.0 fluorometer and an MD enzyme marker.

Sequencing was performed on the Illumina platform, and strict quality control of the data was conducted using fastp software v.23.0 to ensure that the reads were of sufficiently high quality for accurate subsequent analyses [39]. Genes with a false discovery rate (FDR) < 0.01 and |log2Fold Change| ≥ 1 were identified as differentially expressed genes (DEGs), and these transcripts were considered significantly differentially expressed [40]. An enrichment analysis of DEGs was conducted by Gene Ontology (GO) and the Kyoto Encyclopedia of Genes and Genomes (KEGG), with a significance threshold value of *p* ≤ 0.05 [41,42]. DEGs associated with As tolerance and reduction were selected and visualized as heatmaps generated by Sangerbox 3.0 (Hangzhou Mugu Technology Co., Ltd., Hangzhou, China). DEGs encoding functional proteins and transporters, e.g., ATP-binding cassette transporter (ABC), arylsulfatase family member H (ArsH), Arabidopsis response regulators (ARR), and GSHs, were specifically analyzed to investigate As tolerance and reduction mechanisms in *C. cladosporioides*.

Quantitative real-time PCR (qPCR) validation was performed following the method described by Cao et al. [34]. Ten genes and the reference gene *McyG* were used to validate the DEGs identified through Illumina sequencing. The specific qPCR primers for DEGs and reference genes are listed in Table S1.

The Illumina RNA-seq data generated from *C. cladosporioides* have been deposited in the NCBI SRA database under the following accession numbers: SRR32661064, SRR32661063, and SRR32661062 for the three replicates of the control (CK, −As) groups; and SRR32661061, SRR32661060, and SRR32661059 for the three replicates of the As-treated (+As) groups.

### 2.8. Statistical Analysis

Statistical analysis was performed using SPSS 24.0 and Origin 2021 software. Prior to all statistical tests, the assumptions of the normality and homogeneity of variance were verified. Differences among more than two groups or levels under the same variable condition were analyzed using Tukey’s honestly significant difference (HSD) test following a one-way analysis of variance (ANOVA) at a significance level of 0.05. An independent-sample *t*-test was employed to determine the difference between the treatment and control groups at significance levels of 0.05, 0.01, and 0.001. All values are presented as means ± standard deviation (SD) (n = 3). The correlation among DEGs, the total and different valence states of As contents, SOD activities, and the contents of MT, GSH, melanin, MDA, and proline were analyzed using the Mantel test with the linkET R package (https://github.com/Hy4m/linKET, accessed on 10 June 2024) [43]. Pearson correlation analysis was used to assess the relationships between the expression levels of DEGs and the antioxidant system, with a threshold set at r > 0.6 [44]. Graphical analysis was performed using the Origin 2021 software package.

## 3. Results

### 3.1. Taxonomic Identification of Target Fungus

**Figure 1:**
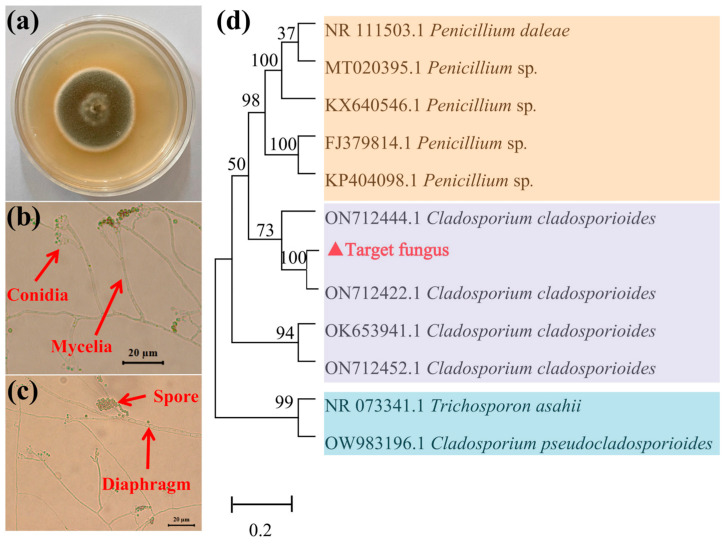
Morphology of the target endophytic fungus colony on PDA medium (**a**), its microstructure (**b**,**c**), and phylogenetic analysis (**d**). The hyphae of the endophytic fungus are septate, with varying numbers of conidia and spores distributed along them. The target strain cluster with *Cladosporium cladosporioides* in a clade with a 100% bootstrap value. Bootstrap values greater than 50% (based on 1000 replicates) are shown above or below the nodes. The scale bar indicates nucleotide substitution in the neighbor-joining analysis. *Trichosporon asahii* and *Cladosporium pseudocladosporioides* were used as outgroup references.

As shown in Figure 1a, the morphological characteristics of the target fungus indicated that the colony color is gray-olivaceous at the center and white to gray-olivaceous at the margins. The colony texture is protuberant at the center and flat at the margins, with diffuse aerial mycelia. As shown in Figure 1b,c, the conidia are straight, solitary, branched, and terminal or lateral, while the mycelium is septate. Based on the Fungal Identification Handbook, the target strain belongs to the phylum Ascomycota, order Dothideomycetes, family Cladosporiaceae, and genus *Cladosporium*.

The ITS rDNA sequences of the target fungus were amplified by PCR and subsequently sequenced with amplicon sizes ranging from 510 to 620 bp. BLAST comparisons revealed 100% homology with the other authentic endophytic fungi sequences previously deposited in GenBank. A total of 11 reference sequences and the target sequence were used to construct a phylogenetic tree. As shown in Figure 1d, molecular identification results indicated that the target filamentous fungus clustered with *Cladosporium cladosporioides* in a clade with a 100% bootstrap value. Based on both morphological and molecular identification results, the target strain was confirmed to be *C. cladosporioides*.

### 3.2. Tolerance of C. cladosporioides to As(V)

**Figure 2:**
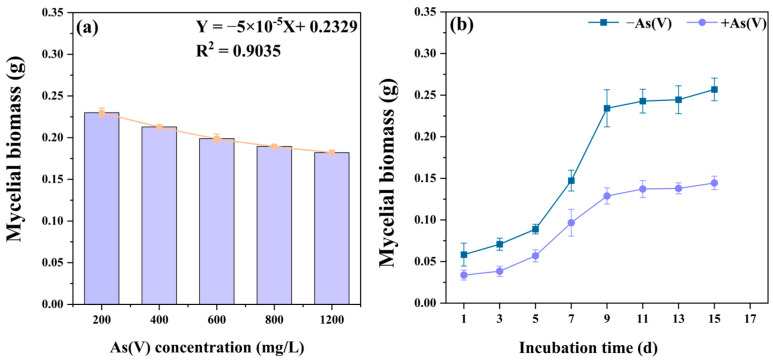
The mycelial biomass and the corresponding linear regressions under different As(V) treatment concentrations (**a**), and the growth curve with and without As(V) treatment (**b**). The linear regression equation is Y = −5 × 10^−5^X + 0.2329, with a correlation coefficient R^2^ of 0.9035. The results demonstrate that increasing As(V) concentrations caused a linear decrease in the mycelial biomass of *C. cladosporioides*, and the EC_50_ values for *C. cladosporioides* resistance to As(V) were as high as 2051.94 mg/L. The growth curve results show that the biomass of the As(V) treatment group was significantly lower than that of the non-As(V) treatment group.

As shown in Figure 2a, increasing As(V) concentrations caused a linear decrease in the mycelial biomass of *C. cladosporioides* within a certain concentration range from 200 mg/L to 1200 mg/L. The EC_50_ values of *C. cladosporioides* resistance to As(V) were calculated by fitting linear regressions to the biomass inhibition results, revealing a high resistance value of 2051.94 mg/L.

To further investigate the growth status, the growth curves of *C. cladosporioides* under As(V) stress and non-As(V) conditions were measured. As shown in Figure 2b, at the same time point, the biomass of the As(V) treatment group was significantly lower than that of the non-As(V) treatment group. However, the growth trends of both groups were similar. A logarithmic growth phase was observed from the third to the ninth day, during which the biomass increased rapidly, followed by a stabilization phase after the ninth day.

### 3.3. Accumulation of Total and Different Valence States of As in Mycelium

**Figure 3:**
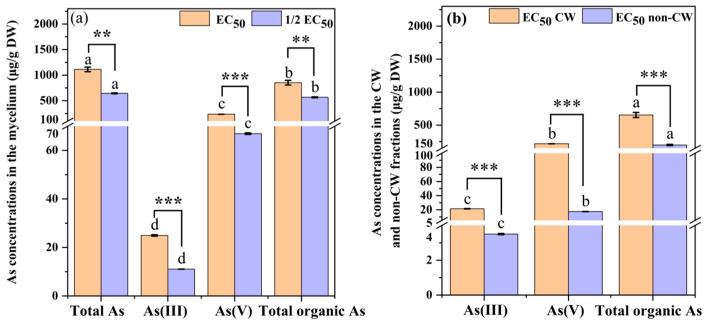
Accumulation of total and different valence states of As in mycelium under the EC_50_ and 1/2 EC_50_ As(V) concentrations (**a**) and their subcellular distribution (**b**). As the As(V) concentration increased, the contents of total As, As(V), and As(III) and total organic As in the mycelium also increased significantly. *C. cladosporioides* can reduce more toxic As(V) to As(III) and organic As, thereby decreasing As toxicity to the cell. Consequently, the proportions of organic As and As(III) reached 76.64% and 2.24%, respectively. The distribution of As(V), As(III), and total organic As in subcellular fractions showed that the content in cell wall fractions was significantly higher than that in non-cell walls, indicating that the cell wall of the mycelium is the primary storage site for As. Different lowercase letters represent significant differences among the contents of different valence states of As, *p* < 0.05. ** and *** represent very significant differences at the levels of *p* < 0.01 and *p* < 0.001, respectively. CW, cell wall fractions; non-CW, non-cell wall fractions.

The contents of total As and As of different valence states were determined to elucidate the tolerance mechanism of *C. cladosporioides.* As shown in Figure 3a, the concentration of total As in the mycelium reached up to 1112.64 μg/g under the EC_50_ As(V) concentration, which was approximately 1.73 times that of the group treated with the 1/2 EC_50_ As(V) concentration. Additionally, as the As(V) concentration increased, the concentrations of As(V), As(III), and total organic As in the mycelium also increased significantly. The reduction of As(V), which is more toxic to microorganisms, to As(III) and organic As is a key detoxification mechanism of *C. cladosporioides*, thereby enhancing As resistance. The concentrations of As(V), As(III), and total organic As in the mycelium were 234.95, 24.94, and 852.75 μg/g, with ratios of 21.12, 2.24, and 76.64%, respectively. Notably, the organic As levels significantly surpassed both As(V) and As(III) concentrations in all cellular fractions (cell wall and non-cell wall components), demonstrating particularly efficient As transformation in *C. cladosporioides*.

Fixing As in the cell wall or storing it in the cytoplasm or vacuole in the form of chelates reduces the oxidative toxicity of free As ions and represents another detoxification mechanism of *C. cladosporioides*. As demonstrated in Figure 3b, the distribution of As(V), As(III), and total organic As in subcellular fractions showed that the content in cell wall fractions was significantly higher than that in non-cell walls. This indicates that the cell wall of the mycelium is the primary storage site for As. In the cytoplasm, the organelles of *C. cladosporioides*, including vacuole, peroxisome, and the endoplasmic reticulum, also play a strong role in the storage and detoxification of As [45]. Notably, the content of total organic As was significantly higher than that of As(V) and As(III) in both the cell wall and non-cell wall fractions under the EC_50_ As(V) concentration. Similarly, the As(V) content is significantly higher than As(III) under the same treatment. This finding is consistent with the distribution pattern of total As.

### 3.4. Changes in the Antioxidant System of C. cladosporioides Under As(V)

**Figure 4:**
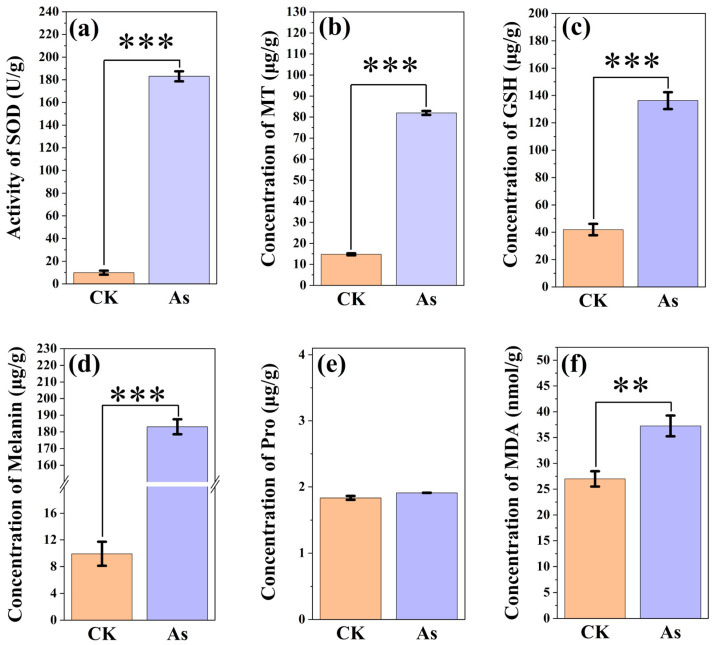
The activity of SOD (**a**) and the contents of MT (**b**), GSH (**c**), melanin (**d**), Pro (**e**), and MDA (**f**) under EC_50_ As(V) concentration. Compared with the non-As treatment, SOD activity and the contents of the MT, GSH, melanin, and MDA increased significantly under the supplementation with the EC_50_ concentration of As(V). This indicates that As(V) caused significant damage to the membrane antioxidant system of *C. cladosporioides*. ** and *** represent very significant differences at the levels of *p* < 0.01 and *p* < 0.001, respectively. SOD, superoxide dismutase; MT, metallothionein; GSH, glutathione; MDA, malondialdehyde; Pro, proline.

SOD is one of the most important enzyme systems for mitigating oxidative damage. As shown in Figure 4a, the SOD activities of *C. cladosporioides* increased significantly by 96.94% (*p* < 0.001) under an EC_50_ concentration of As(V), which helped reduce oxidative damage. Compared to the non-As treatment groups, the contents of MT and GSH in *C. cladosporioides* significantly increased by 454.78% and 224.84%, respectively, under As(V) (Figure 4b,c). Both MT and GSH can readily form complexes with As and be sequestered in vacuoles, thereby reducing oxidative damage. Melanin and Pro, as antioxidants, have the ability to scavenge free radicals and alleviate oxidative damage caused by As stress in microorganisms. Exposure to As(V) significantly increased the concentrations of melanin in *C. cladosporioides* (Figure 4d), while no significant difference was observed in proline content (Figure 4e). The increase in MDA, a common indicator of oxidative stress (Figure 4f), further indicated that As(V) caused significant damage to the membrane antioxidant system of *C. cladosporioides*.

### 3.5. Transcriptome Sequencing and Assembly

The resistance mechanism of *C. cladosporioides* to As at the transcriptional level was revealed through RNA-seq analysis. Two transcriptomes were generated from the groups treated with and without As. Six transcriptomes from the two groups, with three replicates each, yielded a total of 297,647,408 clean reads from 307,354,752 raw reads. The clean data size of each transcriptome was greater than 6.60 Gb. The Q20 and Q30 values for these transcriptomes ranged from 97.98% to 98.41% and 94.19% to 95.12%, respectively, and the overall sequencing error rate ranged from 0.02% to 0.03% (Table S2). Clean reads ranging from 95.14% to 96.08% were mapped to a single locus of the trinity genome using RSEM software 1.3.3 (Table S2). Pearson’s correlation coefficient analysis revealed that the As(V)-treated and non-As group (CK) exhibited a strong correlation (R^2^ ≥ 0.75) between duplicate samples (Figure S1).

### 3.6. Screening and Validation of DEGs and Functional Annotation

**Figure 5:**
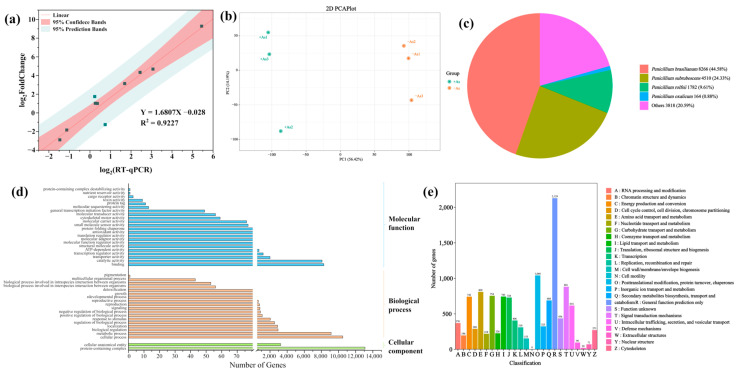
Confirmation of DEGs and functional annotation. (**a**) Validation of RNA-seq DEGs using qRT-PCR. The expression changes in target genes detected by RNA-seq were closely correlated with those determined by qRT-PCR, with a correlation coefficient R^2^ of 0.9227, which confirmed the accuracy and reliability of the RNA-seq results. (**b**) PCA revealed significant differences between the As-treated and non-As-treated groups, with contributions of PC1 and PC2 being 56.42% and 14.19%, respectively. (**c**) Annotated species matching distribution in the *C. cladosporioides* Nr database. More than one-third and one-fifth of unigenes were homologous to the *Penicillium brasilianum* and *Penicillium subrubescens*, respectively. (**d**) GO classification of DEGs. A total of annotated unigenes (approximately 76.57%) of *C. cladosporioides* with BLAST matches to known proteins were assigned to three main GO categories, including biological process (35,122, 47.74%), cellular component (16,320, 22.19%), and molecular function (22,121, 30.07%). (**e**) Based on KEGG and KOG annotation, the DEGs were enriched in pathways such as “metabolic pathways”, “biosynthesis of secondary metabolites”, “carbon metabolism”, “glycolysis/gluconeogenesis”, “lipoic acid metabolism”, and “ABC transporters”.

A total of 4771 DEGs were detected, including 2527 (52.97%) and 2244 (47.03%) upregulated and downregulated genes. Ten DEGs from different gene families were selected for qRT-PCR validation. The expression levels of these DEGs in qPCR and RNA-seq were compared by normalizing FPKM values using log_2_^(Fold_change)^. The results showed that the expression changes detected by RNA-seq were highly consistent with those determined by qPCR, with a correlation coefficient (R^2^) of 0.9227 (Figure 5a), confirming the accuracy and reliability of the RNA-seq results. Principal component analysis (PCA) revealed significant differences between the As-treated and non-As-treated groups, with contributions of PC1 and PC2 being 56.42% and 14.19%, respectively (Figure 5b).

In total, 18,540, 14,476, 15,662, 18,501, and 13,800 unigenes were matched to existing gene models in the Nr, KEGG, Pfam, TrEMBL, and Swiss-Prot protein databases, respectively (Table S3). As illustrated in Figure 5c, among the 20,900 unigenes, 8266, 4510, and 1782 were homologous to *Penicillium brasilianum*, *Penicillium subrubescens,* and *Penicillium rolfsii*, respectively (Figure 5c).

To explore the biological processes associated with these DEGs, GO enrichment analysis was performed. The GO classification results showed that 16,003 annotated unigenes (approximately 76.57%) with BLAST matches to known proteins were assigned to 20 main GO categories. These GO terms were highly relevant to As resistance in cells, including “oxidoreductase activity, acting on paired donors, with incorporation or reduction of molecular oxygen”, “cell wall organization or biogenesis”, “iron ion binding”, “oxidoreductase complex”, and “sulfur compound biosynthetic process” (Figure 5d; Table S4).

The KEGG analysis results showed that 14,476 unigenes (approximately 69.26%) had significant matches in the KEGG pathway database (Table S5). Based on KEGG and KOG annotation, the DEGs were enriched in pathways such as “metabolic pathways”, “biosynthesis of secondary metabolites”, “carbon metabolism”, “glycolysis/gluconeogenesis”, “lipoic acid metabolism”, and “ABC transporters”, which are associated with As resistance in *C. cladosporioides* (Figure 5e; Tables S5 and S6).

### 3.7. Identification of DEGs Involved in the As Resistance of C. cladosporioides

**Figure 6:**
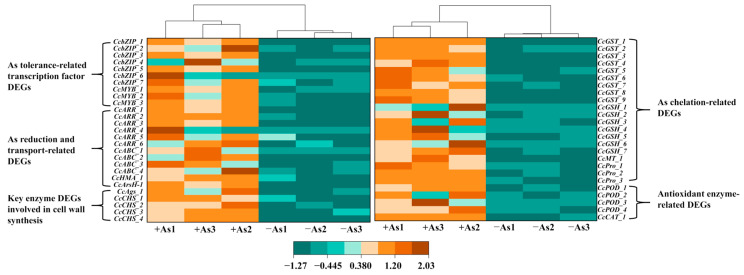
Expression profiles of transcripts involved in As accumulation and detoxification in *C. cladosporioides* under non-As and As conditions. The expression patterns are visualized in a heatmap, constructed using the normalized FPKM value. Data were processed using the Z-score normalization method. The color gradient, transitioning from bottle green to deep yellow, indicates an increase in the expression level. The six boxes in the heatmap, from left to right, represent the values of +As1, +As3, +As2, −As1, −As2, and −As3. The expression levels of DEG, including *CcbZIPs*, *CcMYBs*, *CcARRs*, *CcABCs*, *CcHMA_1*, *CcArsH_1*, *CcAgs_1*, *CcCHSs*, *CcGSTs*, *CcGSHs*, *CcMT_1*, *CcPros*, *CcPODs,* and *CcCAT_1,* were significantly upregulated under As stress. bZIP, basic leucine zipper transcription factors; MYB, v-myb avian myeloblastosis viral oncogene homolog transcription factors; HMA, heavy metal ATPase; Ags, fungal α-glucan synthases; CHS, Chalcone synthase; GST, glutathione-S transferase; POD, peroxidase; CAT, Catalase.

As(V) stress results in a significantly differential expression of a range of genes, including those encoding antioxidant system enzymes or reducing substances, As(V) reductase, As-chelating proteins, and cell wall synthesis pathways. As shown in Figure 6 and Table S7, compared to the non-As group (−As), the expression levels of most DEGs listed in the heatmap were significantly increased under As stress, indicating that the addition of As(V) can activate the cellular antioxidant and defense systems of *C. cladosporioides*. Notably, some of these genes exhibit a remarkable expression range, from undetectable levels to highly elevated levels, such as *CcABC_1*, *CcGSH_1*, *CcGSH_2*, *CcARR_1,* and *CcArsH_1*. DEGs encoding As(V) reductase, such as *CcArsH_1*, *CcARR_1*, *CcARR_2*, and those associated with As absorption and transport proteins, such as ABC, also exhibited upregulated expression. Genes related to cell wall synthesis, such as *CcAgs_1*, *CcCHS_*1, and *CcCHS_2*, were significantly upregulated, as more As was sequestered in the cell walls. Consistent with physiological indices, the expression of genes encoding MT and GSH, and other synthases, such as *CcGSH_1*, *CcMT_1*, and *CcPro_1*, was significantly upregulated under As(V) stress. Additionally, many transcription factor-coding genes exhibit significant differential expression. For instance, *CcbZIP_1*, *CcbZIP_2*, and *CcMYB_1* showed upregulated expression. These findings suggest that these transcription factors may enhance the stress resistance of *C. cladosporioides* by regulating the expression of related genes.

### 3.8. Multiple Correlation Analysis Among As Speciation, Antioxidant System, and Related DEGs

**Figure 7:**
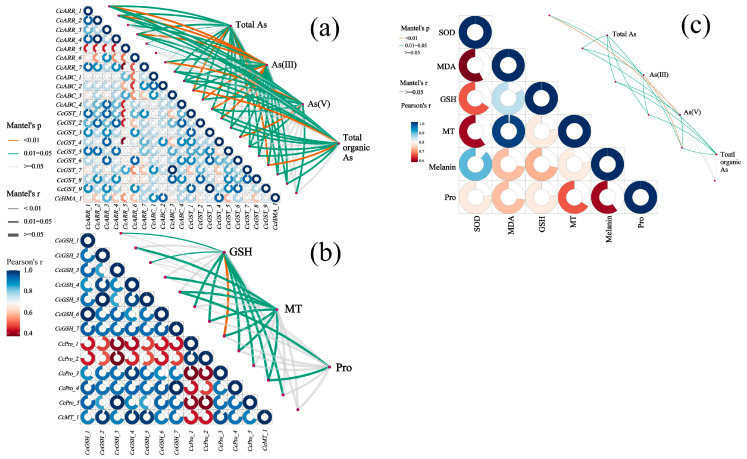
Multiple correlation analysis among As speciation, the antioxidant system, and related DEGs. (**a**) Correlation network between As species (total As, As(III), As(V), and organic As) and functionally annotated DEGs. Significant associations (*p* < 0.01) were observed between DEGs involved in As transport, chelation and reduction pathways, and fungal As speciation profiles (As(III), As(V), and organic As). (**b**) Correlation network between As-chelating compounds and related DEGs. The concentrations of MT, GSH, and Pro, particularly MT and GSH, showed significant positive correlations (*p* < 0.05), with the expression levels of key enzymatic DEGs involved in antioxidant defense and metal chelation pathways. (**c**) Correlation analysis between the antioxidant system and As speciation profiles (total As, As(III), As(V), and total organic As). The concentrations of total As, As(V), As(III), and total organic As exhibited significant positive correlations with the activity of the antioxidant enzyme (SOD) and the levels of antioxidant substances (GSH, MT, and melanin), as well as the oxidative stress marker (MDA). Notably, total organic As showed no significant correlation with either SOD activity or MDA levels (*p* > 0.05), suggesting that organic As species induce substantially less oxidative damage than inorganic As forms. Edge width corresponds to the absolute value of the correlation coefficient determined by the linear mixed-effects models. Colors indicate correlation types. Orange, green, and gray lines denote highly significant (*p* < 0.01), significant (*p* = 0.01−0.05), and non-significant (*p* > 0.05) correlations, respectively, based on the Mantel test. Pairwise comparisons of DEGs or enzyme activities are shown in the triangle, with a color gradient from red to blue (low to high) denoting Pearson’s correlation coefficient.

Figure 7a demonstrates significant Pearson correlations (r > 0.8, *p* < 0.05) between expression levels of As resistance-related DEGs in *C. cladosporioides*, including *CcARR_2*, *CcARR_4*, *CcGST_7*, and *CcABC_3*. Mantel test analysis further revealed strong associations (*p* < 0.01) between these DEGs (functionally annotated in As transport, chelation, and reduction pathways) and fungal As speciation profiles (As(III), As(V), and total organic As). The concentrations of MT, GSH, and Pro, particularly MT and GSH, showed significant positive correlations (*p* < 0.05) with the expression levels of key enzymatic DEGs involved in antioxidant defense and metal chelation pathways (Figure 7b). As shown in Figure 7c, the concentrations of total As, As(V), As(III), and total organic As exhibited significant positive correlations with the activity of the antioxidant enzyme (SOD) and the levels of antioxidant substances (GSH, MT, and melanin), as well as the oxidative stress marker (MDA), particularly for the first three As species. Notably, total organic As showed no significant correlation with either SOD activity or MDA levels (*p* > 0.05), suggesting that organic As species induce substantially less oxidative damage than inorganic As forms.

## 4. Discussion

An expanding body of research demonstrates that root-associated fungi are ubiquitous in plants and play crucial roles in mitigating various biotic and abiotic stresses, including HM toxicity [46]. These fungal symbionts exhibit both remarkable HM resistance and substantial metal accumulation capacity. However, when their host plants are medicinal species cultivated in contaminated soils, this metal enrichment poses significant safety concerns for therapeutic applications. Notable examples include the following: (1) *Epicoccum nigrum* isolated from *Dysphania ambrosioides* (L.) with demonstrated Cd resistance and accumulation [47]; (2) *Cladosporium* sp. and *Cladosporium sphaerospermum* from *Polygonatum kingianum* (Coll. et Hemsl) showing distinct As tolerance and enrichment capabilities [34].

However, research on heavy metal-tolerant endophytic fungi isolated from *G. yunnanensis* remains limited. This medicinal herb, widely used in traditional Chinese medicine (TCM), is predominantly distributed in Yunnan Province. However, its cultivation in metal-rich soils has raised concerns due to reports of excessive HM accumulation in plant tissues [48]. The roots of *G. yunnanensis*, which serve as its medicinal organ, are particularly susceptible to endophytic fungal colonization. We hypothesized that these fungal symbionts may mediate As uptake in the host plant. In this study, one strain with strong As tolerance was screened from the fibrous roots of *G. yunnanensis* grown in a heavy metal-contaminated area. Its morphological characteristics were consistent with the typical structural description of *Cladosporium* [49]. Based on molecular identification results, the As-tolerant strain was identified as *C. cladosporioides*. Evidence indicates that *Cladosporium* is a major root endophytic fungus, rather than a phytopathogen [50]. However, there are limited reports on its As tolerance mechanisms.

The EC_50_ value is widely recognized as one of the key indicators for assessing the susceptibility of microbial tolerance to HMs [51]. In this study, *C. cladosporioides* exhibited high As tolerance, with an EC_50_ value of 2051.94 mg/L. This tolerance threshold significantly exceeded those reported for other As-tolerant fungi: *Phialocephala fortinii* (800 mg/L) [52] and *Gaeumannomyces cylindrosporus* (1860 mg/L) [53]. While *C. cladosporioides* exhibits robust growth capability under As stress in vitro, both biomass production and growth rate showed concentration-dependent inhibition, with significant reductions observed at As(V) concentrations exceeding 200 mg/L (Figure 2a,b). The growth curve of *C. cladosporioides* under As stress was significantly inhibited (Figure 2b). These findings are consistent with those reported previously by Hou et al. [54], in which the biomass of endophytic fungi *Acrocalymma vagum* and *Scytalidium lignicola* declined under increasing Cd stress [55]. The quantification of As accumulation in *C. cladosporioides* revealed extraordinary bioconcentration capacity, with the total As reaching 1112.64 µg/g in mycelia (Figure 3a). This represents a 1.69-fold increase over previously reported values (660 µg/g) in fungal systems [56]. The total As content in the cells was partitioned into cell wall-bound As and non-cell-wall-bound As. The results indicated that the non-cell-wall-bound As content was significantly lower than that in the cell wall, with the former being approximately 4.06 times higher than the latter (Figure 3a). This distribution pattern aligns with established findings in the literature. For instance, Shukla et al. [13] demonstrated that HMs like As preferentially accumulate in microbial cell walls. Furthermore, both cell wall-bound and non-cell-wall-bound As fractions showed concentration-dependent increases in response to elevated As treatment levels (Figure 3b). However, contrasting findings by Mohd et al. [57] revealed an inverse relationship between As concentration and cellular distribution: the proportion of cell wall-bound As increased while non-cell wall fractions decreased with rising As levels. Supporting this observation, Mohd et al. [58] demonstrated that the cell walls of the rice endophytic fungus *Piriformospora indica* exhibit remarkable As adsorption capacity, reaching up to 4 mg/g of fresh cell wall weight. This cell wall sequestration is recognized as the primary As resistance mechanism in this fungal species, where As complexation within the cell wall matrix prevents ion entry and subsequent cellular damage [59]. Similar wall-binding detoxification appears operative in *C. cladosporioides*. Complementing this extracellular strategy, intracellular detoxification occurs through the following: (1) a complexation of As ions with thiol-rich peptides (GSH and MT) and (2) subsequent compartmentalization within vacuoles [60]. This dual-phase defense system—combining cell wall immobilization and intracellular chelation/sequestration—significantly enhances fungal As resistance.

Accumulating evidence indicates that microbial As detoxification primarily occurs through the reduction of As(V) to As(III) and subsequent transformation to organic As species. Our findings demonstrate that *C. cladosporioides* exhibits a particularly efficient As transformation system, with organic As concentrations significantly exceeding both As(V) and As(III) levels in both cellular fractions (Figure 3b). Notably, we observed the following concentration gradient: organic As > As(V) > As(III), suggesting a robust metabolic conversion of highly toxic As(V) to less toxic organic and trivalent forms. This substantial biotransformation capacity directly contributes to the fungus’s enhanced As resistance. These results align with the work of Mandal et al. [61], who reported similar As reduction capabilities in *Microbacterium paraoxydans*, where the reduction pathway was shown to be crucial for As stress tolerance. A common As metabolic pathway found in microbes is methylation. Starting from inorganic As, the As S-Adenosyl methionine (SAM)-dependent methyltransferase (ArsM in microbes) is capable of adding methyl groups to As to form mono-, di-, and tri-methylated arsenicals. The oxidative methylated As species, including methylarsenate (MAs(V)), dimethylarsenate (DMAs(V)), and trimethylarsine oxide (TMAsO), are detected in many organisms and environments [62]. The methylation process is generally regarded as a detoxification pathway because the methylation product is usually organic As with less toxicity [63]. This precisely explains our research results, wherein the *C. cladosporioides* reduces toxicity by metabolizing As(III) and inorganic As into less toxic organic As species through methylation, thereby mitigating their harmful effects on the *C. cladosporioides*.

To mitigate oxidative damage induced by HM toxicity, fungi have evolved sophisticated antioxidant defense systems [64]. When HM ions enter cells, they catalyze the production of reactive oxygen species (ROS), which can oxidatively damage critical biomolecules, including membrane lipids, proteins, chloroplast pigments, enzymes, and nucleic acids [65]. Increasing evidence has shown that SOD, MT, GSH, melanin, MDA, and Pro can be used to uncover the resistance mechanisms that protect cells from oxidative damage [66]. In this study, As(V) stress significantly enhanced SOD activity in *C. cladosporioides* (Figure 5a). These findings align with those of Mukherjee et al. [67], who reported a 9.3-fold increase in SOD activity in *Aspergillus niger* at 25 mg/L of As(V). The elevated SOD activity suggests an adaptive detoxification response to neutralize superoxide radicals generated by As(V) exposure [54].

Under As(V) stress, *C. cladosporioides* exhibited significantly increased MT and GSH levels (Figure 5b,c). These results corroborate previous findings in other fungi: *Laccaria bicolor* showed elevated GSH under Cd/As exposure [68], while *Paxillus involutus* demonstrated increased MT production in response to Cd stress [69]. Both GSH and MT function as dual-purpose defense molecules, acting not only as metal chelators but also as protective agents against metal-induced oxidative damage [60]. Melanin has been widely studied as an antioxidant that enhances HM stress tolerance in microorganisms. It mitigates metal-induced stress by alleviating oxidative damage and activating antioxidant enzymes like SOD [70]. In this study, *C. cladosporioides* exhibited significantly increased melanin production under As(V) stress (Figure 5d). These findings align with research on *Exophiala pisciphila*, a metal-tolerant endophyte isolated from *Arundinella bengalensis* roots, which similarly showed elevated melanin levels under metal stress conditions [71]. While Pro typically serves multiple protective roles under metal stress-functioning as an osmolyte, radical scavenger, electron sink, and stabilizer of cellular structures and macromolecules [72], its concentration in *C. cladosporioides* showed no significant changes under the experimental conditions. MDA, a cytotoxic byproduct of lipid peroxidation, serves as a reliable biomarker for oxidative stress and membrane damage [73]. In *C. cladosporioides*, MDA levels increased significantly following 7-day As(V) exposure, demonstrating HM-induced oxidative damage. This response pattern mirrors observations in *Exophiala* spp. under Cd exposure [30], suggesting a conserved oxidative stress mechanism across metal-tolerant fungi.

To elucidate the molecular mechanism underlying As tolerance, we performed transcriptome sequencing to analyze DEGs in *C. cladosporioides* under As(V) stress. Numerous studies have shown that stressors trigger a large number of genes and proteins, thereby activating signaling pathways that confer stress tolerance [74]. Transcriptomic analysis revealed 4771 DEGs in *C. cladosporioides*, comprising 2527 upregulated and 2244 downregulated genes. This indicates that *C. cladosporioides* underwent significant changes in growth, reproduction, and resistance defense mechanisms to adapt to As stress. The DEGs were primarily associated with cell wall organization or biogenesis, iron ion binding, metabolic pathways, the antioxidant system, ABC transport, the biosynthesis of secondary metabolites, and As reductase. (Figure 5). These enriched functional categories were directly involved in *C. cladosporioides* As resistance mechanisms. Our findings align with previous observations in *Sphingomonas desiccabilis*, where As stress similarly induced the differential expression of genes related to the following: (1) cell wall biogenesis; (2) iron ion homeostasis; (3) secondary metabolite production; (4) antioxidant defense systems; (5) ABC transporter activity [75]. The upregulation of ABC transporters represents a conserved HM detoxification strategy across species. For instance, *Exophiala pisciphila* showed 26 Cd-responsive ABC transporter DEGs [76], whereas our study identified 19 As(V)-inducible ABC transporter genes. This consistent pattern demonstrates that ABC transporter induction is a fundamental microbial adaptation to metal stress, functioning to reduce intracellular metal accumulation, maintain cellular viability, and restore metabolic homeostasis [77].

Interestingly, DEGs exhibited a broad spectrum of expression levels, spanning from undetectable to highly elevated (Figure 6; Supplementary Table S7). This pattern mirrors observations in *Neurospora crassa*, where *CHR-1* expression was significantly upregulated under Cr stress but undetectable in control conditions [78]. Key DEGs in *C. cladosporioides,* including *CcGSH_1*, *CcGSH_2*, *CcPro_1*, *CcARR_1*, *CcARR_2*, *CcGST_1*, and *CcABC_3*, encode critical components of As resistance, such as GSH, MT, Pro, arsenate reductases, ABC transporters, and cell wall organizers. Their expression levels strongly correlated with the physiological markers of As stress, including total/valence-specific As accumulation and GSH/MT concentrations (Figure 7). Similar gene-to-phenotype linkages have been reported in *Symphytum officinale* under Zn exposure [79] and *Fusarium proliferatum*/*Aspergillus terreus* under Ni and Cd stress [80]. These consistent findings across species underscore that the physiological adaptations to HMs stem from coordinated gene expression changes, collectively mitigating oxidative damage and enhancing stress resistance.

Research has demonstrated that endophytic fungi not only regulate their own antioxidant systems and molecular mechanisms to resist HM stress but also significantly enhance plants’ tolerance to HMs. For instance, Rahman et al. found that the endophytic fungus *P. indica*, isolated from *Artemisia annua* L., exhibits high tolerance to As. Notably, As accumulation was significantly higher in the roots than in the shoots of *P. indica*-inoculated *A. annua*. This suggests that As immobilization in the roots is directly associated with successful fungal colonization, which restricts As translocation to aerial parts [81]. Therefore, *C. cladosporioides* can be highly recommended as a promising candidate for phytostabilization, helping mitigate the adverse effects of heavy metals on plants in contaminated environments.

## 5. Conclusions

This study provides novel insights into the As tolerance mechanisms of *C. cladosporioides* isolated from the fibrous roots of *G. yunnanensis*, integrating physiological and molecular analyses. (1) High As tolerance: *C. cladosporioides* exhibited exceptional As tolerance, with an EC_50_ value of 2051.94 mg/L based on biomass analysis. (2) Subcellular distribution and redox transformation: Significant As accumulation occurred in the mycelium, with cell wall-bound As concentrations of 4.06-fold higher than non-cell wall fractions. The total concentrations of As, As(V), As(III), and organic As were predominantly localized in cell wall fractions. This distribution pattern indicates that cell wall sequestration serves as the primary detoxification strategy in these fungi. The metabolic conversion of highly toxic As(V) to less toxic As(III) and organic As was observed, alongside the activation of antioxidant systems (SOD, MT, GSH, melanin). (3) Transcriptomic adaptations: As stress induced 4771 DEGs (2527 upregulated; 2244 downregulated), enriched in pathways, including cell wall biogenesis, iron ion binding, ABC transport, secondary metabolite biosynthesis, and arsenate reductase activity. Many DEGs were uniquely and highly expressed under As stress, such as *CcARR_1* and *CcArsH_1*. (4) Integrated defense strategy: strong correlations linked physiological markers (total organic As/As species and MT/GSH/proline levels) with DEG expression. Taken together, *C. cladosporioides* exhibits strong As enrichment characteristics and can effectively enhance its resistance to As stress by reducing the highly toxic As(V) to the less toxic As(III) and organic As, improving the membrane antioxidant system and upregulating the expression of DEGs involved in transporter, reductase, and chelating protein. These findings highlight *C. cladosporioides* as a promising candidate for As-contaminated soil remediation and elucidate its role in As accumulation within *G. yunnanensis*.

## Data Availability

The data presented in this study are deposited in the National Center for Biotechnology Information (NCBI) Sequence Read Archive database (SRA) with an accession number of PRJNA1234892.

## Supplementary Materials

The following supporting information can be downloaded at: https://www.mdpi.com/article/10.3390/jof11050374/s1. Figure S1. Sample correlation analysis. Clustering heatmap revealed strong reproducibility between biological replicates (R^2^ ≥ 0.75) for both As-treated (+As) and control (−As/CK) groups. Table S1. qPCR primer pairs used for transcriptome validation. Table S2. Sequencing data quality metrics for *C. cladosporioides* libraries. Table S3. Functional annotations of *C. cladosporioides* unigenes across five protein databases. Table S4. Gene Ontology (GO) classification of unigenes. Table S5. KEGG pathway enrichment of unigenes. Table S6. KOG classification of unigenes. Table S7. FPKM values of DEGs in *C. cladosporioides*. Table S8. DEGs selected for Mantel test analysis.
