# Supplementary material for: Arsenic Stress Resistance in the Endophytic Fungus Cladosporium cladosporioides: Physiological and Transcriptomic Insights into Heavy Metal Detoxification
Source: J Fungi (Basel). 2025 May 14;11(5):374. doi: 10.3390/jof11050374 (PMC12112881; doi:10.3390/jof11050374)
Supplement: Supplementary file 1 [file jof-11-00374-s001.zip › jof-3614616-supplementary/Supplementary Table S1-S3 R 3 30.pdf]

Table S1 Primer pairs used for qPCR in the confirmatory test of transcriptome

Serial number	Name of genes	Sequences Primer (5′-3′)
1	*CcArsH_1*	F:CATTCACTCAACACGGCTTCTC<br>R:TGTAGGCTATGCGTTTCTCG
2	*CcARR_1*	F:GTCCCCGCACAGCAAACT<br>R:TCCGCCTTCCAGAATCAA
3	*CcGSH_1*	F:GCAAATATGCGGCAAAGGAG<br>R:AGACGAGACGATTAGGTGGG
4	*CcGSH-2*	F:GATGGTGGCTGCGACTACA<br>R:CGGCAGCAACACCAATAAC
5	*CcGST-1*	F:TCATTAGTGGAGTAGACGGACG<br>R:TTGCTGCGGAGAAAGTGC
6	*CcGST_2*	F:TCATCGGTTGTCGGGTGT<br>R:CGTCGCCAACTCGTGTATTA
7	*CcGST_3*	F:GTTCATCAAGGTCATCAGCC<br>R:CGAAGTAGTAACCAGCCAAG
8	*CcABC_1*	F:AAACGTCTCACTGTGACCTTCC<br>R:TGTCCTTTTGCGGTTCTCCT
9	*CcABC_2*	F:AGGATCGCCAGACGAAGAAG<br>R:AAGCACATTGAGCAGGGTTG
10	*CcNIP_1*	F:GCGATTCTCGTTACGCCTCT<br>R:CAGCCCAGTTTACCCAGTGTT
11	Reference genes (*McyG*)	F:CATTACAAGTGACCCCGGTCTAAC<br>R:CCCCGGAGGCAACAGAG

Table S2 Summary of data output quality of various *C. cladosporioides* libraries

Groups	Sample names	Raw reads	Clean data						Mapped reads(%)
			Reads	Bases(G)	Errors(%)	Q20(%)	Q30(%)	GC (%)	Total
	+As_1	45,893,426	44,463,574	6.67	0.02	98.13	94.42	53.16	21,199,608(95.36%)
+As	+As_2	56,272,766	53,436,692	8.02	0.02	98.41	95.12	53.21	25,418,954(95.14%)
	+As_3	454,27,288	44,020,104	6.60	0.02	98.12	94.38	53.19	20,991,711(95.37%)
	-As_1	53,108,620	51,623,220	7.74	0.02	98.04	94.36	54.63	24,780,320(96.00%)
-As	-As_2	53,926,944	52,510,100	7.88	0.03	97.98	94.19	54.61	25,179,760(95.90%)
	-As_3	52,725,708	51,593,718	7.74	0.02	98.05	94.37	54.61	24,785,166(96.08%)

Table S3 Functional annotations of *C. cladosporioides* in five protein databases

Database	Number of Unigenes	Percentage (%)
Annotated in Nr	18,540	88.71
Annotated in KEGG	14,476	69.26
Annotated in Pfam	15,662	74.94
Annotated in Trembl	18,501	88.52
Annotated in Swiss-Prot	13,800	66.03
Annotated in at least one Database	18,892	90.39
Total Unigenes	20,900	100
