# Supplementary figures and images for: Arsenic Stress Resistance in the Endophytic Fungus Cladosporium cladosporioides: Physiological and Transcriptomic Insights into Heavy Metal Detoxification
Source: J Fungi (Basel). 2025 May 14;11(5):374. doi: 10.3390/jof11050374 (PMC12112881; doi:10.3390/jof11050374)

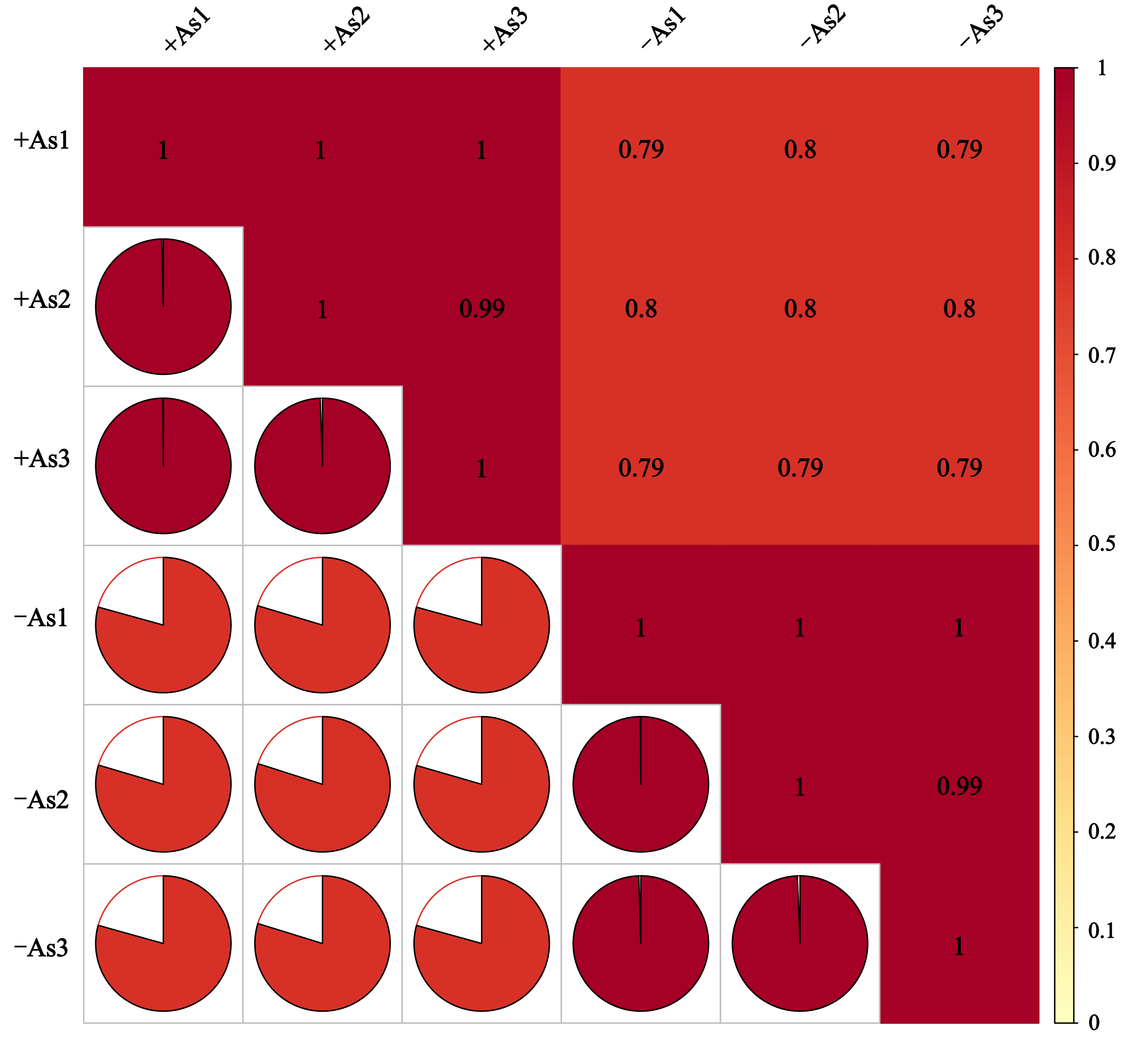

Supplement: Supplementary file 1 [file jof-11-00374-s001.zip › jof-3614616-supplementary/Figure S1.tif]
